# Mass media exposure and its impact on malaria prevention behaviour among adult women in sub-Saharan Africa: results from malaria indicator surveys

**DOI:** 10.1186/s41256-018-0075-x

**Published:** 2018-07-04

**Authors:** Sanni Yaya, Olalekan A. Uthman, Agbessi Amouzou, Ghose Bishwajit

**Affiliations:** 1 School of International Development and Global Studies, University of Ottawa, 120, University Private, Ottawa, ON K1N 6N5 Canada; 2 Warwick Centre for Applied Health Research and Delivery (WCAHRD), Division of Health Sciences, Warwick Medical School, University of Warwick, Coventry, CV4 7AL UK; 3 Bloomberg School of Public Health, Johns Hopkins University, 615 N Wolfe St, Baltimore, MD 21205 USA

**Keywords:** Malaria prevention behaviour, Insecticide treated nets, Antimalarial drugs, Global health, Sub-Saharan Africa

## Abstract

**Background:**

Mass media exposure plays a pivotal role in health communication and adoption of a healthy lifestyle. In this study, we aimed to measure the prevalence of malaria prevention behaviour among adult women in eight malaria-endemic countries in sub-Saharan Africa (SSA), and assess the influence of mass media exposure in the adoption of those behaviours.

**Methods:**

For this study, we collected cross-sectional data on 46,822 women aged between 15 and 49 years from the Malaria Indicator Surveys (MIS) conducted in Burkina Faso, Ghana, Mali, Malawi, Kenya, Nigeria, Sierra Leone and Uganda. As the outcome variable, malaria prevention behaviour was proxied by the use of insecticide treated nets (ITNs) and uptake of antimalarial drugs in last pregnancy.

**Results:**

The overall prevalence of sleeping under ITN and that of taking antimalarial drug during the last pregnancy was respectively 67.9% (95%CI = 66.6–69.2) and 72.8% (95%CI = 71.3–74.2). However, there were disparities in the prevalence of using ITN and antimalarial drug use across the study countries. In the multivariable regression analysis, not receiving malaria related information from radio, poster/billboards, community events, and health workers were found to be significantly associated with reduction in the odds of using ITN the previous night. For the use of antimalarial drugs during last pregnancy, the odds were 23% [OR = 0.773, 95%CI = 0.625–0.956] lower for those who did not receive malaria information on radio compared with those who received.

**Conclusions:**

These findings indicate a potentially important role of malaria information received through mass media on utilisation of ITN among women in SSA. More research is needed to explore the factors that limit the accessibility to malaria information through mass media.

## Background

In Africa, malaria costs approximately $12 billion in terms of loss of Gross Domestic Product (GDP) each year, and has slowed the pace of economic growth by 1.3% per year as a result of lost lives and low productivity [1]. In 2012, an estimated 207 million people suffered from this preventable and treatable disease, with about half of the global population still at risk of transmission [2]. There is large disparity in the pattern of malaria; while the global malaria prevalence indicates that increasing number of countries in Asia and South America are being able to approach elimination [3], countries in sub-Saharan Africa on the other hand still remain the most endemic region accounting for about 90% of the global malaria-related deaths [4].

Malaria burden is most entrenched among the poorest communities who often lack adequate knowledge about the causes, consequences and capacity to afford treatment and adopt preventive measures [5, 6]. Added to the affordability and behavioural issues, are the demographic and gender dimensions which are usually less frequently talked about [6]. There is compelling evidence regarding the fact that children and women are more vulnerable to malaria-related morbidity and mortality [6–8] with pregnant women about twice more likely to be at risk of transmission [9]. Besides the direct consequences, malaria during pregnancy (gestational malaria) is also an important contributor to maternal anaemia which is associated with higher risk of pregnancy complications and adverse birth outcomes [10–12]. Given the long-term consequences of gestational malaria on maternal and child health, the World Health Organization recommends several preventive tools including the use of insecticide treated nets (ITNs) and provision of antimalarial drugs (e.g. sulfadoxine-pyrimethamine intermittent preventive treatment (SP-IPTp)) during prenatal health check-ups [10]. According to a systematic review, using ITNs is an important mode of prevention that has beneficial effects against parasite prevalence in all gravidities in the endemic regions of Africa [13]. Recommended uptake of SP-IPTp has also shown to improve maternal haemoglobin count and decrease the prevalence of placental malaria infection at delivery and low birth weight [14–16].

Despite the demonstrated benefits of these preventive measures, the use of antimalarial drugs and ITNs remains far lower than optimum [14, 17, 18]. While the national and international alliances are developing strategies to increase the affordable provision of these services among women in Africa [1], the efficacy and success of these efforts will ultimately depend on their adequate utilisation which is largely reliant on women’s awareness of the challenges and knowledge of prevention. The healthcare system also has a vital role to play in reducing the persistently high rates of malaria and associated morbidities in SSA and to promote effective health communication, health literacy and treatment-seeking-behaviour among the population [19, 20]. Lack of knowledge and awareness regarding a disease is a major drawback to prevention and intervention of any public health challenge; this can be addressed greatly by strengthening health communication tools and disseminating health information through the conventional and/or digital media [21–23].

Multi-dimensional approaches have been employed to promote the use and uptake of malaria prevention and treatment. In Nigeria, mass media campaigns have been used for sensitization, especially among pregnant women on the benefits of regular ITN use [21]. The print and electronic media have been prominently used in behavioural change communications in many developing countries [21, 23]. Other forms of communication in the community such as health literacy (through community drama, religious institutions), paper (poster, billboard, newspaper) and digital (television, radio) media have been found to serve as important vehicles for the transmission of health knowledge and have been the subject of researches as public health behaviour modification tools [5, 6, 21]. However, the evidences are sporadic for African countries and not much is known about the influence of mass media on malaria prevention behaviour such as the uptake of antimalarial drug use in pregnancy and the use of ITNs. In the light of the above, we conducted this study using data collected in Burkina Faso, Ghana, Mali, Malawi, Kenya, Nigeria, Sierra Leone and Uganda between 2014 and 2016, to investigate the impact of exposures to mass media on malaria prevention among women.

## Methods

### Settings and data source

**Fig. 1:**
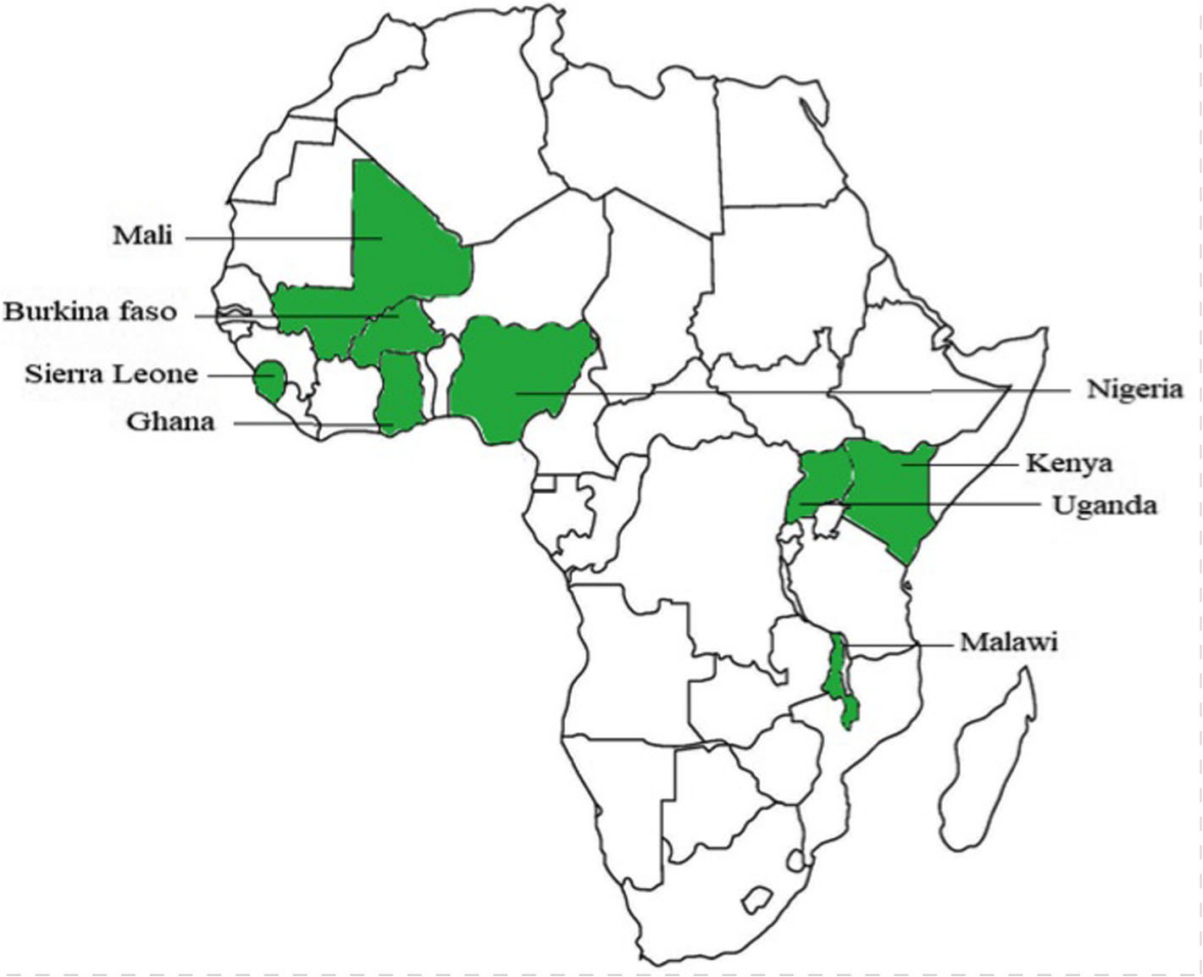
Countries included in the study. Shows the Sub-Saharan African countries included in the study. Eastern (Kenya, Malawi and Uganda) and Western (Burkina-Faso, Ghana, Mali, Nigeria and Sierra Leone)

Data were obtained from the MIS conducted in Burkina Faso (2014), Ghana (2016), Mali (2015), Malawi (2014), Kenya (2015), Nigeria (2015), Sierra Leone (2016), Uganda (2014–15). The surveys were conducted with technical assistance from the National Bureau of Statistics (NBS) of each country. It was funded by ICF International through the Demographic and Health Survey (DHS) Program which is also a USAID supported project in the implementation of population and health surveys [24]. The countries were selected based on geographical diversity. The SSA countries included in this study were thus; Eastern (Kenya, Malawi and Uganda) and Western (Burkina-Faso, Ghana, Mali, Nigeria and Sierra Leone) countries respectively (Fig. 1) [25].

The MIS s employed a stratified two-stage cluster design. In the first stage, sampling strata were created from which clusters were selected by a probability-proportional-to-size technique. A complete listing in the selected clusters served as the sampling frame for the second stage. In the second stage, households were selected from each cluster by equal probability systematic sampling. Study participants were women aged 15–49 years residing in non-institutional places in urban and rural areas. Details of the survey procedure have been reported in the method section of a related MIS report [26].

The surveys were to provide quality data for measuring the progress of effective monitoring and assessing national malaria programs implementation. Specifically, the surveys focused on measuring the indicators of ownership and use of mosquito bed nets, coverage of the intermittent preventive treatment programme for pregnant women, treatment-seeking behaviour, measuring indicators of knowledge and attitudes related to malaria control, and determining the factors associated with malaria and anaemia. Besides these, the survey also captured women’s basic demographic and educational information, information on use of ITNs, malaria prevention practices during pregnancy, and knowledge of malaria. Further details on survey are available from the data sources [24].

### Variables

The outcome variables were two self-reported indicators of malaria prevention behaviour among the participants: 1) Sleeping under ITN the previous night, and 2) Use of antimalarial drugs in last pregnancy. Both of the questions were answered with the options of ‘Yes’ and ‘No’.

The independent variables of interest were reports of receiving malaria information through mass media and interpersonal communication. The following sources reported by the participants were included in the analysis and were categorised as ‘Yes’ or ‘No’: TV, Radio, Religious institutions, Poster, Community event, Health worker.

To measure the independent associations of malaria prevention behaviour, the analysis was adjusted for some potentially confounding variables selected through evidence on their correlation with the outcome and explanatory variables. The following were found relevant to the analysis: Age: 15–19/ 20–24/ 25–29/ 30–34/ 35–39/ 40–44/ 45–49; Setting: Urban/ Rural; Education: No education/ Primary/ Secondary/ Higher; Religion: Islam/ Christian/ Other; Wealth index: Poorest/ Poorer/ Middle/ Richer/ Richest.

All the variables were self-reported except for the wealth index. For the calculation of household wealth status, the volume of durable goods (e.g. TV, radio, bicycle) possessed by the household as well as housing quality (e.g. type of floor, wall, and roof) were taken into consideration. Each item was assigned a factor score generated through principal component analysis (PCA) which were then summed and standardized for the households. These standardised scores placed the households in a continuum based on relative wealth scores. The scores obtained were categorized into quintiles to rank the household as poorest/poorer/middle/richer/richest [27].

### Data analysis

Statistical analyses were performed with SPSS version 24. Firstly, the datasets were cleaned for outliers, checked for potential multicollinearity, and then merged to perform pooled analysis. Due to nonproportional allocation of the samples across the survey regions, they were not self-weighted. Furthermore, MIS surveys use cluster sampling design. To adjust for sample weight and cluster sampling techniques, the analysis was preceded by the preparation of complex survey file by accounting for primary sampling units, sample strata and sample weight. Prevalence of the outcomes variables for each explanatory variables were presented in percentages. In addition, the percentage use of ITN and antimalarial drug used during pregnancy was presented for each study country. Multivariable binary logistic regression model was used to calculate the odds ratios of the associations between ITN use and uptake of antimalarial drug for the last pregnancy with mass media exposure and interpersonal communication channels. Each outcome variable was regressed as a function of exposure to the six different types of mass media and to the binary logistic regression, while adjusting for various demographic and socioeconomic variables which were selected based on standard conceptual framework [28]. Results of regression analysis were presented as odds ratios along with their 95%CIs as an indicator of significance as well as precision of the OR values. For all associations *P*-value of < 0.05 was considered statistically significant.

### Ethical approval

The protocol of DHS surveys was approved by the Ethics Committee of ORC Macro Inc. [16, 17]. The study was based on analysis of anonymized secondary data available in the public domain of DHS, hence, no further ethical approval was necessary for the study.

## Results

### Description of sample population

In total 46,822 women aged between 15 and 49 years (Mean = 28.32) were included in the present study. Country-wise percentage distribution of participants were as follows: Burkina Faso (17.3%), Ghana (10.9%), Sierra Leone (9.9%), Mali (16.6%), Uganda (11.0%), Kenya (11.3%), Nigeria (17.0%), and Malawi (6.0%). The sociodemographic and mass media exposure information are presented in Table 1.

**Table 1:** Distribution of sample population across the explanatory variables (sociodemographic and source of malaria information)

	*N* = 46,822	% (95%CI)	Slept under ITN last night (67.9, 95%CI = 66.2–69.2)	Took antimalarial drug in last pregnancy (72.8, 95%CI = 71.3–74.2)
Age group				
Mean = 28.32 (SD 9.04)				
15–19	9093	19.2%, (18.5–19.9)	17.5%, (16.7–18.2)	7.3%, (6.9–7.8)
20–24	8797	18.3%, (17.8–18.9)	18.6, (18.0–19.2)	22.4%, (21.7–23.2)
25–29	8662	18.8%, (18.3–19.2)	19.4%, (18.8–19.9)	25.7%, (25.0–26.5)
30–34	7240	15.6%, (15.1–16.1)	15.9%, (15.3–16.5)	21.4%, (20.7–22.1)
35–39	5934	13%, (12.5–13.5)	13.7%, (13.0–14.3)	14.7%, (14.1–15.2)
40–44	4137	8.8%, (8.5–9.2)	8.8%, (8.4–9.2)	6.4%, (5.9–6.8)
45–49	2959	6.3%, (6–6.5)	6.3%, (6.0–6.5)	2.1%, (1.9–2.4)
*p*-value			< 0.001	0.047
Setting				
Urban	15,083	34.1%, (26.5–42.7)	71.2%, (61.5–79.2)	70.0%, (67.3–72.6)
Rural	31,739	65.9%, (57.3–73.5)	28.8%, (20.8–38.5)	30.0%, (27.4–32.7)
*p*-value			< 0.001	< 0.001
Education				
No education	19,771	39.6%, (35.6–43.6)	7.0%, (6.0–8.2)	3.7%, (3.2–4.3)
Primary	10,247	22.8%, (21.0–24.6)	22.7%, (20.1–25.5)	23.0%, (21.6–24.4)
Secondary	11,740	28.5%, (25.7–31.4)	23.1%, (21.0–25.3)	22.7%, (21.5–23.9)
Higher	5064	9.2%, (7.9–10.7)	47.2%, (43.2–51.3)	50.6%, (48.7–52.6)
*p*-value			0.003	< 0.001
Religion				
Islam	21,935	50.1%, (44.4–55.8)	49.0%, (42.0–56)	49.5%, (47.2–51.8)
Christian	16,608	35.5%, (30.6–40.8)	37.9%, (31.8–44.3)	32.1%, (30.2–34.0)
Other	8279	14.3%, (11.4–17.8)	13.1%, (10.4–16.5)	18.5%, (16.7–20.3)
*p*-value			< 0.001	< 0.001
Wealth index				
Poorest	9720	20.9%, (16.7–25.8)	17.7%, (11.2–26.9)	18.6%, (17.3–19.9)
Poorer	9182	18.3%, (16.2–20.7)	18.1%, (16.0–20.4)	20.0%, (18.9–21.2)
Middle	9274	19.4%, (17.5–21.4)	20.3%, (18.2–22.6)	21.0%, (19.9–22.1)
Richer	9253	19.8%, (17.6–22.3)	20.5%, (18.1–23.1)	20.4%, (19.1–21.8)
Richest	9393	21.6%, (15.5–29.2)	23.4%, (18.7–28.8)	20.0%, (18.0–22.0)
*p*-value			0.019	< 0.001
Typesof media to receive malaria information				
TV				
No	38,801	84.1%, (79.1–88.1	84.9%, (78.70–89.6)	78.6%, (76.9–80.1)
Yes	8021	15.9%, (11.9–20.9	15.1%, (10.4–21.3)	21.4%, (19.9–23.1)
*p*-value			< 0.001	< 0.001
Radio				
No	26,267	56.1%, (54.7–57.4)	48.2%, (46.8–49.6)	46.5%, (45.0–48.1)
Yes	20,555	43.9%, (42.6–45.3)	51.8%, (50.4–53.2)	53.5%, (51.9–55.0)
*p*-value			< 0.001	< 0.001
Religious institutions				
No	42,233	90.2%, (89.1–91.1)	19.7%, (18.6–21.9)	31.9%, (30.8–32.1)
Yes	4589	9.8%, (8.9–10.9)	80.3%, (78.1–81.4)	68.1%, (67.9–69.2)
*p*-value			< 0.001	< 0.001
Poster				
No	43,113	91.4%, (89.6–93.0)	9.7, (7.7–12.1)	11.1%, (9.9–12.3)
Yes	3709	8.6%, (7.0–10.4)	90.3, (87.9–92.3)	88.9%, (87.7–90.1)
*p*-value			< 0.001	< 0.001
Community event				
No	43,477	92.4%, (90.6–93.9)	9.4, (7.4–11.8)	11.1%, (10.0–12.3)
Yes	3345	7.6%, (6.1–9.4)	90.6, (88.2–92.6)	88.9%, (87.7–90.0)
*p*-value			< 0.001	< 0.001
Health worker				
No	35,354	74.4%, (71.6–77.1	33.1, (30.2–36.3)	63.2%, (61.4–65.0)
Yes	11,468	25.6%, (22.9–28.4	66.9, (63.7–69.8)	36.8%, (35.0–38.6)
*p*-value			< 0.001	< 0.001

N.B. *p* significant at < 0.05

The overall prevalence of sleeping under ITN in the night preceding the interview was 67.9% (95%CI = 66.6–69.2), and that of taking antimalarial drug during the last pregnancy was 72.8% (95%CI = 71.3–74.2). Table 1 further shows that the prevalence rates of both of these indicators tended to be higher among women aged 25–29 years, residing in urban areas, having higher level of education, followers of Islam, living in non-poor households, and women who reported getting malaria related information through television, radio, religious institutions, posters, community events and health workers.

**Fig. 2:**
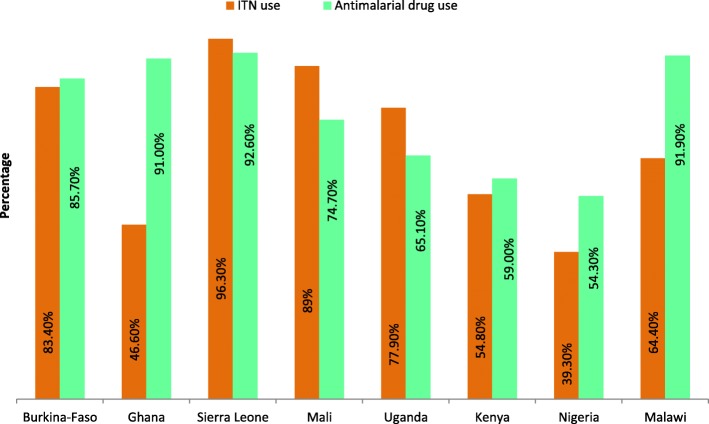
ITN and antimalarial drug use among women. Shows the percentage utilization of ITN and antimalarial drug during pregnancy among women

The percentage utilization of ITN and antimalarial drug during pregnancy were presented in Fig. 2. The percentage use of ITN was less than 40% in Nigeria (39.3%). While Ghana and Kenya also reported low use of ITN accounting for 46.6 and 54.8% respectively. However, other countries which had improved use of ITN include Sierra Leone (96.3%), Mali (89.0%) and Burkina-Faso (83.4%). Furthermore, antimalarial drug use during previous pregnancy was slightly above 90% in Sierra Leone, Malawi and Ghana respectively. Nonetheless, Nigeria, Kenya and Uganda accounted for 54.3, 59.0% and about two-third respectively. These results showed large disparities in ITN and antimalarial drug use during pregnancy across various SSA countries.

Multivariable analysis on the association between mass media exposure and interpersonal communication with malaria prevention behaviour.

According to the results of multivariable regression presented in Table 2, participants who reported not receiving malaria related information from radio, poster/billboards, community events and health workers had respectively 36% [OR = 0.643, 95%CI = 0.593–0.699], 43% [OR = 0.572, 95%CI = 0.466–0.704], 52% [OR = 0.483, 95%CI = 0.394–0.59], and 56% [OR = 0.441, 95%CI = 0.395–0.493] significantly lower odds of using ITN last night compared with those who reported receiving malaria information from these sources. For the use of antimalarial drugs during last pregnancy, the odds were 23% [OR = 0.773, 95%CI = 0.625–0.956] lower for those who did not receive malaria information on radio compared to those who received.

**Table 2:** Association between social media use status and malaria prevention behaviour among women aged 15–49 years in selected sub-Saharan countries

	Slept under ITN last night		Took antimalarial drug during pregnancy	
	Unadjusted OR (95% CI)	Adjusted OR (95% CI)	Unadjusted OR (95% CI)	Adjusted OR (95% CI)
See/hear messages about malaria on local TV				
Yes	1.034	1.041	1.034	1.467
No	(0.843–2.244)	(0.979–1.359)	(0.843–1.944)	(0.988–2.170)
Hear messages about malaria on the local radio				
Yes	0.546 *	0.643 *	0.546 *	0.773 *
No	(0.507–0.588)	(0.593–0.699)	(0.507–0.888)	(0.625–0.956)
Hear messages about malaria in the worship places				
Yes	0.525 *	1.041	0.525 *	0.825
No	(0.452–0.609)	(0.889–1.218)	(0.452–0.609)	(0.465–1.464)
Have seen or heard messages on a poster or billboard				
Yes	0.653 *	0.572 *	0.653 *	1.106
No	(0.539–0.792)	(0.466–0.704)	(0.539–0.792)	(0.697–1.753)
Have seen or heard messages at a community event				
Yes	0.399 *	0.483 *	0.399 *	1.297
No	(0.329–0.485)	(0.394–0.591)	(0.329–0.485)	(0.788–2.136)
Hear messages about malaria with health worker/NGOs				
Yes	0.235 *	0.441 *	0.235 *	0.896
No	(0.213–0.259)	(0.395–0.493)	(0.213–0.259)	(0.642–1.250)
Nagelkerke R-Squared	0.175	0.372	0.175	0.530

N.B. Yes = reference category. ^*^ = Significant at *p* < 0.05. Adjusted OR = Adjusted for Age, setting, education, religion, wealth status, country

## Discussion

### Main findings

The findings of this study showed that there is need for improvement in using ITN regularly and taking antimalarial drugs during pregnancy. Striking variances were observed in the prevalence of ITN use among the eight countries. While Sierra Leone, Mali, Burkina Faso, attained coverage of over four-fifth among the adult women population, the situation seems to be far more challenging for other countries including Nigeria, Ghana, Kenya, and Malawi where about two-fifth to three-fifth of the women are not using ITNs. Similarly, some countries are clearly far behind than others in achieving optimum coverage for utilisation of antimalarial drug during pregnancy.

Except for women aged 15–19 years and antimalarial drug use, younger women were more likely to use both the services compared to those aged 40 years and above. The increase in antimalarial drug use during pregnancy could be as a result of the fertility level among the young women, which is generally known to outscore the older counterpart. Again, it is probable that the younger generations are enjoying a higher exposure to mass media and health related information, which could help them to become more aware of their health needs and environmental conditions. Women in the rural areas, those with low educational qualification and poor households were found to be less adherent to malaria prevention behaviour. Majority of the African population are living in rural areas where grid connectivity is still a serious problem, and more so the communities remain deprived of the benefits of health communication offered by fast expanding digital media technologies [22]. As the results further describe, television and radio were the most commonly reported sources of malaria information after direct communication with healthcare workers.

Percentages of women sleeping under ITN and taking antimalarial drugs in last pregnancy were higher among participants who reported receiving malaria information from TV, radio, poster/billboard, religious institutions, community events and healthcare workers. In the model that adjusted for potential confounders, receiving malaria information through radio, poster, community event and health worker turned out to be significant predictors of using ITN. However, not receiving malaria information through the mass media including radio amongst others, significantly resulted to reduction in the odds of ITN and antimalarial drug use during pregnancy.

### Previous studies

Country representative studies on the use of ITN among women are still scarce, however there are evidences of appreciable success in increasing ITN coverage in the general population for several countries. In Burkina Faso, for instance, household ownership of ITN has increased dramatically from 5.6 to 89.9% between 2003 and 2014, a success that is largely attributable to the free mass distribution of ITNs in the years 2010 and 2013 [29]. At sub-national levels the prevalence of ITN use varies from 56.3% in Western Kenya [30], 90% in Malawi [31], and 27% in northern Nigeria [17]. These variations are hard to account for in light of the present analysis. Some possible explanations might include socioeconomic inequalities and the coverage and efficacy of the respective programs. For example, although Mali introduced its first IPTp and ITN policy (in 2003 and 2006 respectively) only a few years after Kenya (in 1999 and 2001 respectively) [32], a considerable disparity can be seen in the prevalence of ITN and IPTp use between these two countries.

In the current literature the evidence on the association between mass media exposure malaria prevention practices are scarce. However, studies in Cameroon [33], Gambia [34], Ghana, Malawi Nigeria [22], highlighted the importance of targeting the dissemination of health knowledge through traditional media. In Gambia, songs performed by community members were found to be effective in encouraging people to repair bed nets [34].

### General discussion and policy implications

In the last 10–15 years tremendous efforts have been made to attain universal coverage of ITN and gestational use of antimalarial drugs, the two most cost-effective tools of prevention of malaria. Several countries in SSA have received free ITN campaign and distribution, thereby succeeded in increasing household ownership of ITNs. However, there are evidences on the gap between ownership and optimum use of the preventive services [35], addressing which could help the programs attain the full potential of the services being provided [36]. Achieving the universal coverage and utilisation goal of ITN and IPTp, can be greatly enhanced by improving people knowledge and awareness about malaria and encouraging adoption of preventive behaviour among the population. Social media strategies have proven to be useful in promoting health knowledge among adult populations in all settings, and can be leveraged by malaria elimination programs in Africa as well. Health policy making should focus on empowering women with health knowledge as it can help women develop their own perceptions and knowledge base of the particular disease and enable them to better communicate their health issues with physicians.

For long-terms success, the broader macroeconomic conditions e.g. research and funding, should also be given special attention. As more countries are progressing towards malaria elimination, the donor financing malaria programs has also been declining since 2010 [37, 38]. Many malaria-eliminating countries have projected national declines in funding from the Global Fund to Fight AIDS, Tuberculosis, and Malaria (GFATM), which has been the largest financial supporter of malaria since 2002 [39]. The changing priority of donor agencies can put the countries in SSA in a difficult situation with respect to their pursuit of malaria elimination. Since these are the least wealthy of all countries who are experiencing the highest incidence, it is very unlikely that national funding alone will be able to keep up with intervention and elimination efforts. National leaders in Africa need to gather efforts to reduce dependency on foreign donors and develop regional partnership for addressing local challenges.

### Strengths and limitations of the study

Among the strengths of the present study is the large sample size, generalizability of the findings, women aged 15–49 years, and comparative presentation of the data that allows understanding of the situation in a particular country relative to others. However, there are several limiting points including the secondary nature of the data which means that authors had no control over the measurement and selection of the variables. Although the incidence of malaria is perennial in some regions, for certain there is a factor of seasonality. Therefore, not being able to account for high or low transmission period of the surveys might have affected the prevalence of ITN use. For instance in Kenya, the prevalence of ITN use was found to be significantly higher during the rainy season compared to that in dry season [30]. Apart from the seasonal variation in ITN use, another potential confounder for the prevalence might be the use of unobserved protective behaviour or mechanisms e.g. better housing environment, insecticide sprays, and traditional methods. Information on antimalarial drug use for the last pregnancy is subject to some degree of recall error. We were also unable to deduce the variation in the predictability of different types of mass media (digital, paper, health worker) on ITN use and uptake of antimalarial drug. There was no information on the exact contents of the messages women received through the above mentioned sources. Lastly, data were cross-sectional and therefore no causal relationship can be made from our analysis.

## Conclusion

The present study aimed to investigate the situation of malaria prevention behaviour among adult women in selected countries in sub-Saharan Africa. The findings revealed that the prevalence of ITN use and antimalarial drug uptake among pregnant women remains inadequate. This situation calls for strengthening the current efforts to improve the coverage and utilisation of the preventive measures. To this regard, better distribution and social marketing of ideas are required which might be facilitated by developing an innovative health communication strategy for the social media. National and regional malaria intervention programs can benefit from utilising existing knowledge on the role and efficacy of mass media exposure on malaria prevention behaviour among women. Despite many limitations, the findings provide a contrasting scenario on the usage of ITNs among individual women and antimalarial drugs among pregnant women, which are expected to assist in tracking progress of the current programs and for scaling up malaria elimination strategies in the studied countries and beyond. Future studies should aim to understand the impact of communicating the best practices through mass media, as well as the factors that may influence people’s acceptance of or trust on health-related information gained from public channels. More in-depth and qualitative studies are also required to explore the sociocultural aspects of malaria prevention behaviour.

## Abbreviations

CI: Confidence interval
DHS: Demographic Health Survey
GFATM: Global Fund to Fight AIDS, Tuberculosis, and Malaria
ITNs: Insecticide treated nets
MIS: Malaria Indicator Surveys
OR: Odds ratio
PCA: Principal component analysis
SP-IPTp: Sulfadoxine-pyrimethamine intermittent preventive treatment
SSA: Sub-Saharan Africa
